# Individual participant data validation of the PICNICC prediction model for febrile neutropenia

**DOI:** 10.1136/archdischild-2019-317308

**Published:** 2019-11-05

**Authors:** Bob Phillips, Jessica Elizabeth Morgan, Gabrielle M Haeusler, Richard D Riley, On behalf of the PICNICC Collaborative

**Affiliations:** 1 Centre for Reviews and Dissemination, University of York, York, UK; 2 Leeds Children's Hospital, Leeds, UK; 3 Infectious Diseases and Infection Control, Peter MacCallum Cancer Centre, Melbourne, Victoria, Australia; 4 Research Institute for Primary Care and Health Sciences, Keele University, Keele, UK

**Keywords:** haematology, infectious diseases, oncology, statistics

## Abstract

**Background:**

Risk-stratified approaches to managing cancer therapies and their consequent complications rely on accurate predictions to work effectively. The risk-stratified management of fever with neutropenia is one such very common area of management in paediatric practice. Such rules are frequently produced and promoted without adequate confirmation of their accuracy.

**Methods:**

An individual participant data meta-analytic validation of the ‘Predicting Infectious ComplicatioNs In Children with Cancer’ (PICNICC) prediction model for microbiologically documented infection in paediatric fever with neutropenia was undertaken. Pooled estimates were produced using random-effects meta-analysis of the area under the curve-receiver operating characteristic curve (AUC-ROC), calibration slope and ratios of expected versus observed cases (E/O).

**Results:**

The PICNICC model was poorly predictive of microbiologically documented infection (MDI) in these validation cohorts. The pooled AUC-ROC was 0.59, 95% CI 0.41 to 0.78, tau^2^=0, compared with derivation value of 0.72, 95% CI 0.71 to 0.76. There was poor discrimination (pooled slope estimate 0.03, 95% CI −0.19 to 0.26) and calibration in the large (pooled E/O ratio 1.48, 95% CI 0.87 to 2.1). Three different simple recalibration approaches failed to improve performance meaningfully.

**Conclusion:**

This meta-analysis shows the PICNICC model should not be used at admission to predict MDI. Further work should focus on validating alternative prediction models. Validation across multiple cohorts from diverse locations is essential before widespread clinical adoption of such rules to avoid overtreating or undertreating children with fever with neutropenia.

**What is already known on this topic?**

- Validation of prediction models is uncommonly performed beyond the first description of a model.
- Febrile neutropenia can be managed in a risk-adapted way, but the best method of risk prediction is unclear.
- The Predicting Infectious ComplicatioNs In Children with Cancer (PICNICC) model produces risk estimates of infection in febrile neutropenia from clinical information and routine blood tests.

**What this study adds?**

- This meta-analytic study showed across seven studies with 1159 patients the prediction model performed variably but poorly.
- Recalibration using three different simple approaches did not overcome the poor results.
- The original PICNICC model is not reliable in predicting risk of infection in children presenting with febrile neutropenia and should not be used in practice.

## Background

The side effects of cancer therapies in childhood frequently require unplanned admission to hospital with consequent heavy burden on patients, families and the health service. The most common side effect leading to such an admission is the suspicion of severe infection in an immunocompromised child, known as fever with neutropenia. This is experienced by most patients at least once,^1^ and is associated with a median hospital stay of 5 days.^2^

The management of fever with neutropenia commonly consists of admission to hospital and the delivery of intravenous antibiotics to minimise adverse outcomes such as death or disability. This approach produces low mortality rates^2^ but overtreats the 75% of individuals who do not have a documented infection.^1^ The potential for adverse consequences includes the emergence of resistant microorganisms^3^ and secondary hospital-acquired infections^4^ which have been associated with prolonged antibiotic exposure and hospitalisation. Inpatient therapy is also associated with inferior health-related quality of life for children with cancer^3^ and outpatient options are often chosen where available.^4^ Personalising an approach to fever with neutropenia could be achieved by (1) facilitating those who wished to be discharged to go home if predicted to be at a low risk of serious infection,^5^ and (2) by using biomarkers of infection/inflammation to identify those in whom it is appropriate to shorten the duration of antibiotic therapy.^6^

A risk prediction model has been developed by the ‘Predicting Infectious ComplicatioNs In Children with Cancer’ (PICNICC) collaboration.^7^ This international collaboration, which included 24 groups from 16 countries, derived a new clinical model to predict the risk of infection in febrile neutropenic episodes from nine of these data sets. This joins four other models which appear to have reasonable validity or applicability in this population.^8^ The model was developed using a strong internal validation process, including shrinkage techniques, cross-validation (by leave-one-out techniques) and bootstrapping to guard against overfitting and inadequate performance in new data sets. However, it requires external validation, as previous studies have found that initial descriptions of risk prediction models may be overly optimistic in new data and can perform differently in different settings.^9 10^

This study aimed to externally validate the predictive performance (ie, discrimination and calibration of risk predictions) and clinical utility of the model developed by the PICNICC Collaboration, by collecting and analysing data from multiple geographically diverse cohorts of children and young people who had developed fever with neutropenia. Estimates of predictive performance are summarised using meta-analysis, and the variations between data sets examined to identify differences in performance across different units and countries. Our goal was to establish whether the model is globally robust, or if it was only suitable in particular settings. This is important in clinical practice as previous work has suggested that despite broad international consensus on the therapies used in treating childhood cancer, important between-continent variation occurs in the accuracy of FN decision rules.^8^

## Methods

### Study design

Individual participant data (IPD) meta-analysis of existing cohort studies to externally validate a previously derived prediction model.

Studies were included of cohorts of children and young people receiving anticancer treatment who developed fever and neutropenia, or who presented clinically septic and afebrile/hypothermic after such treatment. Each study was required to provide sufficient anonymised information to calculate the PICNICC prediction model with appropriate outcomes. Studies were sought through invitations within the PICNICC network, and the permission to share the information sought by the originating team.^11^ Outcomes were defined according to international consensus recommendations^12 13^ with microbiologically documented infection (MDI) defined as an infection that was clinically detectable and microbiologically proven, and bacteraemia isolation of a recognised pathogen cultured from one or more blood cultures or common commensals cultured from two or more blood cultures from separate occasions.

### Sample size

We aimed to collect IPD that, in total across studies, had at least 100 events (ie, MDIs) as this is the minimum recommended for external validation of a risk prediction model for a binary outcome.^14^

### Method of analysis

External validation of the performance of a risk prediction model consists of two components: statistical validation of predictive performance and clinical utility.^15^

First, to summarise predictive performance, the discrimination and calibration performance of the model’s predictions were calculated separately for complete cases in each data set and then summarised across studies using random-effects meta-analysis with the restricted maximum likelihood estimator and inverse variance weighting.^16 17^ In each study separately the calibration and discrimination characteristics were calculated. Calibration, the agreement between the model’s predicted risks and the observed risks across individuals, was quantified by calculating the ratio of expected to observed cases (E/O ratio, ideal value of 1). Discrimination is the ability of the model’s predicted risks to correctly separate those who will and those will not develop the episode, examined by the separation of predicted risks on the calibration plot and quantified by the area under the receiver operating characteristic curve (AUROC, also known as the C-statistic), with values closer to 1 showing higher discrimination. Estimates of variance for each estimate were derived from bootstrapping with replacement, using 2000 separate draws.

The performance estimates across all studies were then summarised by random-effects meta-analysis, for each of the AUROC, calibration slope and E/O ratio separately. A random-effects meta-analysis accounts for unexplained between-study heterogeneity in predictive performance, which is expected.^17 18^ The summary (pooled) results describe the average predictive performance, and the between-study heterogeneity is measured by ‘tau-squared’ (larger values indicate greater heterogeneity) and a 95% prediction interval (PrI), which describes the model’s expected performance in a new setting.^16^

The predictive performance of the model was also re-evaluated after strategies for recalibration of the intercept and/or calibration slope. Recalibration is expected as the occurrence of complications may differ from the derivation set,^18^ such that the baseline risk or values of the coefficients need to be tailored to the local population.^19^ The choice of recalibration approach remains an ongoing matter of investigation,^20^ and we compared four approaches as follows. The first three approaches changed the model intercept, but kept the same predictor effects (ie, the beta coefficient values) as in the original model. The baseline recalibration approach (A) used a weighted average intercept from the derivation IPD, assuming the different study-level intercepts in the development data were drawn from a normal distribution. Then alternatives based on study-specific estimates were used: (B) basing the estimated intercept on the proportionate rate of MDI, and (C) interpolating the intercept from meta-regression of the intercept on the proportion of MDI. Lastly, approach (D) modified the beta coefficient values of the original model by multiplying them by the calibration slope observed in the new data and using the same intercept calculated as the average across all studies.^14^ Meta-analysis was used to summarise and compare after each recalibration strategy.^20^

Finally, after undertaking these recalibration approaches, exploratory analyses were undertaken to re-evaluate the beta coefficient values of the model variables to determine causes of inaccurate estimation.

Clinical utility was assessed by calculating the sensitivity (Sn) and specificity (Sp) of dichotomising at ≤10% chance of serious complications initially, and comparing this with estimates from the derivation data set.^16^ This calculates how many patients would be categorised as ‘low risk’ and what proportion of this group developed a serious complication. Sn and Sp were summarised across studies using bivariate meta-analysis technique, with data derived from each of the raw and recalibrated approaches.

The article is reported according to the Transparent Reporting of a multivariable prediction model for Individual Prognosis or Diagnosis (TRIPOD) guidelines, and all analyses were done using R V.3.2.0.

## Results

### Included studies

Six study groups provided IPD from seven independent data sets, and these were included in the initial validation (see table 1). The invitation to join the PICNICC Collaborative, and the processes of acquiring and validating the data, are detailed in other publications.^7 21^ Those providing data for the validation submitted their information after the derivation had commenced, and so were kept aside for this project.

**Table 1:** Properties of the data sets

	Episodes, n	Patients, n	MDI episodes, n	MDI (%)	Study design
Leeds	48	27	9	19	Prospective
Liverpool	47	21	7	21	Retrospective
Sheffield	167	47	51	31	Retrospective
Nottingham	121	63	41	26	Retrospective
Belgium	27	16	5	19	Prospective
Melbourne a	101	54	18	18	Retrospective
Melbourne b	648	327	154	24	Retrospective

MDI, microbiologically documented infection

The populations in the validation studies varied in their geographical origin, and demographics of the included patients (see table 2, and online supplementary appendix 1 for distribution of the linear predictors).

10.1136/archdischild-2019-317308.supp1 Supplementary data

**Table 2:** Demographic outline of the patients in the data sets

	Leukaemia (%)	Lymphoma (%)	Solid tumours (%)	Brain tumours (%)	Median age (min-max)	Outpatients (%)	Poststem cell transplant (%)	Central line in situ (%)
Leeds	11 (41)	3 (11)	10 (37)	3 (11)	67 months (15–219)	41 (85)	2 (7)	48 (100)
Liverpool	9 (43)	2 (9)	10 (48)	0	70 months (8.5–228)	21 (100)	0	21 (100)
Sheffield	19 (40)	4 (9)	17 (36)	7 (15)	79 months (5–205)	150 (84)	15 (9)	161 (98)
Nottingham	37 (59)	1 (1.5)	19 (30)	6 (9.5)	48 months (6–118)	155 (100)	4 (6)	141 (91)
Belgium	9 (56)	2 (13)	5 (31)	0	86 months (30–277)	0	1 (4)	24 (89)
Melbourne a	63 (62)	10 (10)	24 (24)	4 (4)	6 years (1–14)	101 (100)	0	62 (96)
Melbourne b	180 (55)	25 (8)	88 (27)	34 (10)	76 months (6.7–241)	648 (100)	15 (4)	310 (95)

### Summarising the predictive performance of the original model

**Figure 1:**
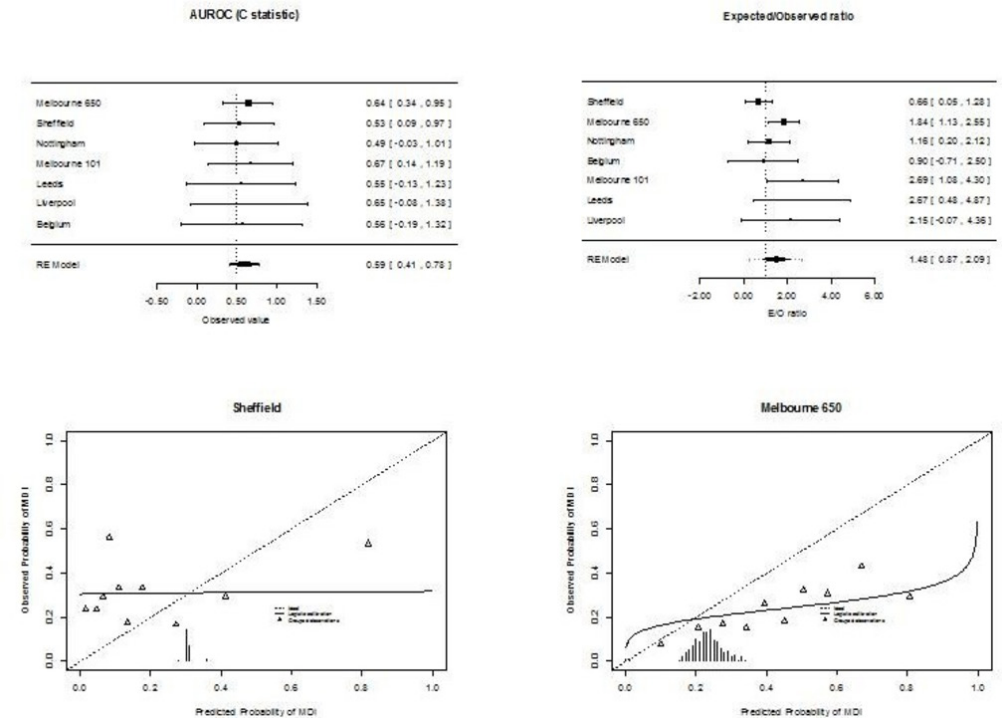
Meta-analytic analysis of the performance of the original Predicting Infectious ComplicatioNs In Children with Cancer (PICNICC) model. The forest plots demonstrate the values obtained from each data set, and their pooled summary value. The calibration plots demonstrate how the predicted probability from the PICNICC score matches the observed proportion of MDI; ideal calibration sits along the diagonal from bottom left to top right, indicated by the dotted line. AUROC, area under the receiver operating characteristic curve; E/O, expected/observed ratio; MDI, microbiologically documented infection.

Using the weighted average intercept and coefficients from the derivation model showed a lower C-statistic than the derivation model (validation pooled C-statistic of 0.59, 95% CI 0.41 to 0.78, tau^2^=0, compared with original model C-statistic of 0.72, 95% CI 0.71 to 0.76). This was related to a systematic overestimation of the risk of MDI: pooled E/O ratio 1.48, 95% CI 0.87 to 2.1, 95% PrI 0.26 to 2.28, tau^2^=0.21, with poor calibration (pooled slope estimate 0.03, 95% CI −0.19 to 0.26, tau^2^=0). See figure 1 for forest plots of C, E/O and exemplar calibration plots. Online supplementary appendix 2 contains individual calibration plots.

### Predictive performance after recalibration attempts

Using alternative intercepts, either a proportionate change in the baseline MDI rates (strategy b) or by interpolation of the meta-regression estimates (strategy c), both on a study-by-study basis, led to almost identical values (see online supplementary appendix 3).

**Figure 2:**
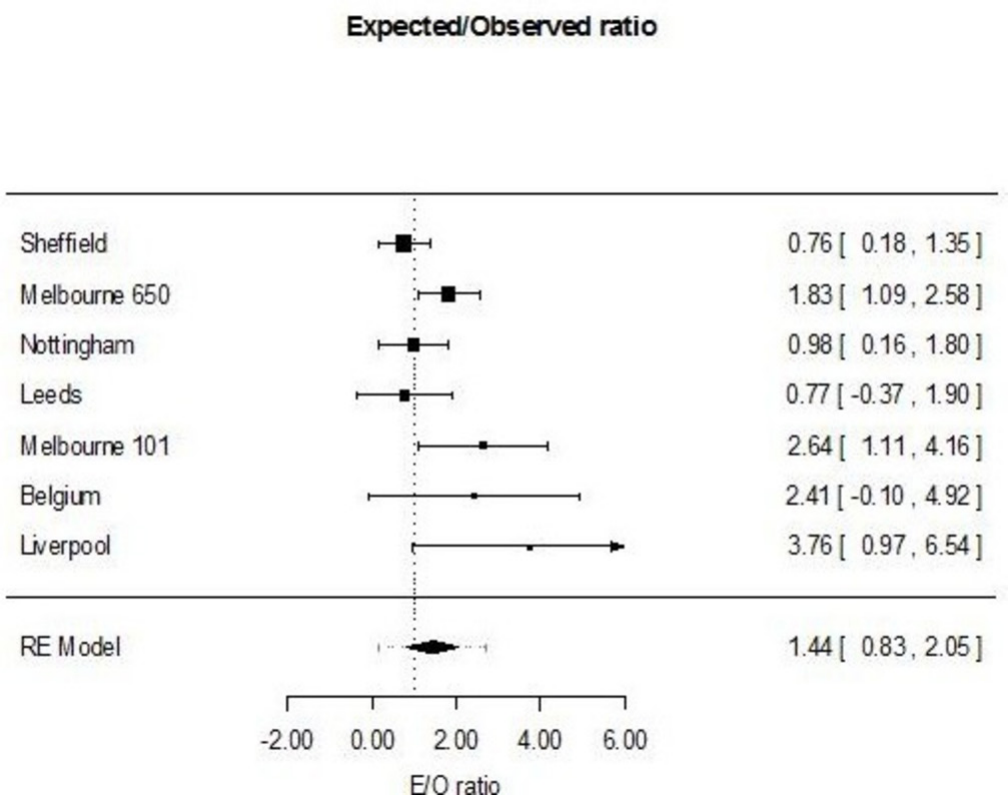
Meta-analytic analysis of the performance of the Predicting Infectious ComplicatioNs In Children with Cancer (PICNICC) model after study-specific slope recalibration. E/O, expected/observed ratio; RE, random effects.

Altering the calibration slope based on the new data and using the intercept derived from the meta-analysis of all the validation data (strategy d) did not alter the rank order of the linear predictor, and so the C-statistic did not change, and each slope was set to 1 by design. The risk of MDI remained significantly overestimated (pooled E/O ratio 1.44, 95% CI 0.83 to 2.05, 95% PrI 0.18 to 2.70, tau^2^=0.31, see figure 2).

### Summary of clinical utility

**Figure 3:**
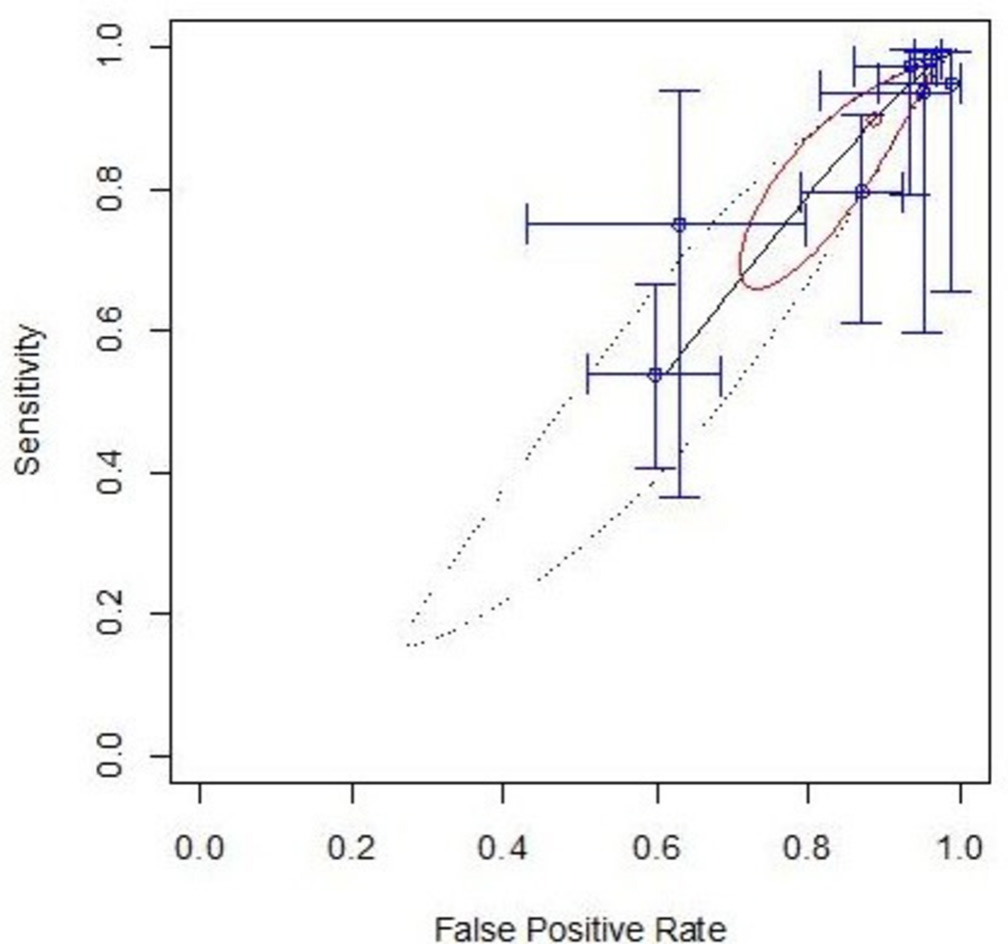
Meta-analytic analysis of the discriminatory performance of the original model in receiver operating characteristic curve (ROC) space. These plots show how sensitivity and specificity are related, with each individual data set producing a cross-hair marking and the pooled summary as the red dot with its confidence region outlined in bold red ellipse, and prediction interval as a dashed ellipse. An ideal test would sit in the top left corner of the plot.

Dichotomising the original model predicted values at a threshold of 10% (into low and high-risk groups), and performing a bivariate meta-analysis of Sn and Sp led to a pooled Sn of 90% (95% CI 72% to 97%) and pooled Sp of 13% (95% CI 5% to 24%), as demonstrated on the ROC space plot (figure 3). Recalibration approaches altering the intercept interpolated from the derivation data (strategy c) led to no meaningful difference in this finding.

**Figure 4:**
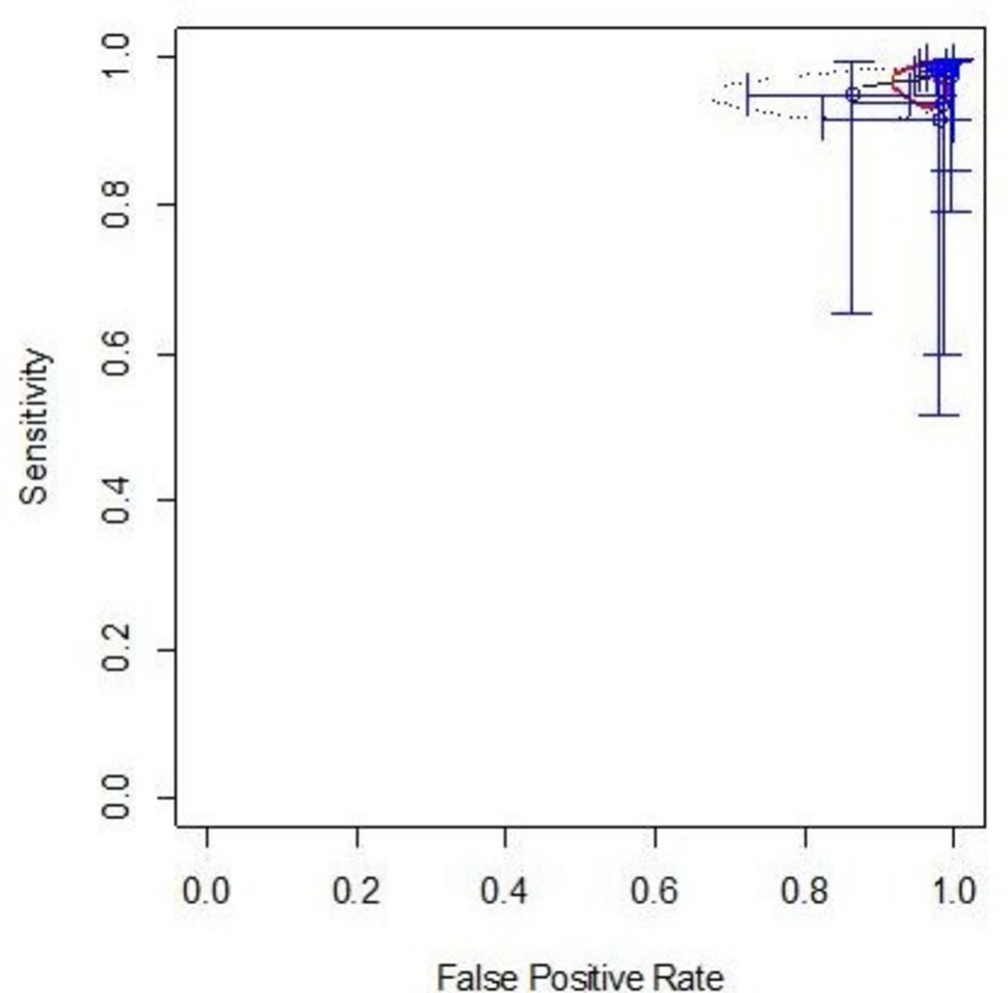
Meta-analytic analysis of the discriminatory performance of the recalibrated model in receiver operating characteristic curve (ROC) space.

Recalibration using the intercept modified by the proportion of MDI (strategy b) led to a deterioration in Sp (pooled values: Sn 91%, 95% CI 69.9% to 97.8%; Sp 7%, 95% CI 1.9% to 31.4%). The study-specific slope and meta-analysis intercept (strategy d) led to an improvement of Sn with a reduction in Sp making the rule effectively useless in practice (pooled values: Sn 97.5%, 95% CI 94.5% to 99.0%; Sp 2.2%, 95% CI 0.7% to 6.6%; identified 20/1115 or 2% of all cases as ‘low risk’; see figure 4).

### Exploratory analysis to understand poor performance

To understand the reason for poor predictive performance of the original model, we re-estimated the model beta coefficients in the validation data. The values are the natural log of the OR of the risk of MDI for each variable; a negative number indicates a decreased risk of MDI, a positive number an increased risk. The SE indicates the uncertainty in the beta coefficient; higher numbers indicate greater imprecision. This analysis produced estimates that differed considerably from those in the original model development, for most of the predictors including for tumour type (excluding the rare and extremely uncertain estimates for germ cell tumour, Hodgkin’s lymphoma, Langerhans cell histiocytosis, retinoblastoma and ‘other sarcoma’, but also rhabdomyosarcoma), temperature, white cell parameters and the gestalt estimate of ‘seriously clinically unwell’ (see table 3).

**Table 3:** Differences in beta coefficients (log ORs) of predictors included in the development model, when calculated in the derivation data set and then in the validation data set

Item	Derivation cohort		Validation cohort		
	Beta estimate	SE of estimate	Beta estimate	SE of estimate	Episodes, n
Acute myeloid leukaemia	0.65	0.26	0.31	0.36	52
Ewing’s sarcoma	−0.64	0.66	−1.08	0.42	71
Germ cell tumour	−0.07	0.88	−13.14	72.64	5
Hepatoblastoma	0.48	0.57	1.04	0.66	12
High-grade brain tumour	−0.34	0.46	0.32	0.3	80
Hodgkin’s lymphoma	−0.41	0.7	−1.07	1.07	13
High-risk neuroblastoma	0.92	0.66	−0.35	0.34	78
Langerhans cell histiocytosis	−14.1	1025.44	1.01	1.27	3
Low-grade brain tumour	−14.16	677.94	0.24	0.47	26
Neuroblastoma	0.47	0.49	−16.44	83.28	2
Non-Hodgkin’s lymphoma	−0.47	0.32	−0.14	0.33	69
Osteosarcoma	−1.19	0.57	0.18	0.33	57
Other tumour	0.8	0.77	−0.93	0.78	17
Retinoblastoma	0.55	0.86	1.05	0.93	5
Rhabdomyosarcoma	−0.24	0.32	−0.18	0.35	67
Sarcoma	0.19	0.82	18.92	115.14	2
Wilms tumour	−0.49	0.66	0.59	0.41	37
Temperature (per °C from 37)	0.57	0.14	0.18	0.14	1152
Clinical impression of ‘Severely unwell’	0.79	0.19	1.29	0.27	1152
Haemoglobin (per g/dL)	0.18	0.05	0	0.06	1152
Natural log (total white cell count)	−0.3	0.1	−0.16	0.09	1152
Natural log (absolute monocyte count)	−0.21	0.06	0.01	0.06	1152

## Discussion

The evaluation of a previously proposed prediction model is an essential but often neglected part of understanding how risk stratification may be implemented in clinical practice. Where radiation therapies are subject to extensive quality assurance and control processes, diagnostic tests marketed with stringent precision, and pharmaceuticals require detailed trials and regulatory agreements, structural service changes, such as the use of prediction models, can be thrown into the clinical arena on minimal evidence. The use of a meta-analytic approach to evaluating a prediction model means it can be tested across multiple environments, enhancing the generalisability of the conclusions which can be drawn.

This analysis shows the initial model as derived by from a global international collaborative was not reliable at predicting which patients who present with episodes of febrile neutropenia have an MDI (pooled C-statistic 0.59, 95% CI 0.41 to 0.79, compared with 0.72, 95% CI 0.71 to 0.76 from the derivation group). No approach to recalibration was effective at resolving the poor predictive performance. The cause of this difference appeared to be in overestimates of predictive ability from the derivation data, when compared with the validation data. These also varied importantly between the sources of data, implying a lack of consistency to the estimates. Clinically, this means the model was only just better in saying which children were going to be diagnosed with an MDI than flipping a coin, despite using a series of simple statistical approaches to correct the estimates. Reducing antibiotic therapy to the PICNICC ‘low risk’ group may undertreat, and pre-emptive increases in antibiotic intensity or coverage in the ‘high-risk’ group would overtreat.

The strength of this study is in its wide range of different locations under analysis, unpublished data sources reducing the many challenges in publication bias, collection of data using a consistent definition guide from the PICNICC group, clear relevance to routine clinical practice and the ability to explore the reasons for miscalibration by re-estimation of the prediction model. It is novel in evaluating a prediction model, as compared with a dichotomising ‘rule’-based approach such as the MASCC^22^ (multinational association of supportive care in cancer) or SPOG^23^ (Swiss paediatric oncology group) systems. There remain limitations imposed by the subjective interpretations of the treating physicians, the varied approaches to diagnosis and therapy which remain between centres and may hide its effectiveness under specific circumstances and the homogenisation of different treatments into a ‘malignancy type’ variable to predict the risk of infection.

A key limitation of this validation may be the choice of ‘microbiologically documented infection’ as the detected outcome. A previous single-centre validation of the PICNICC rule^24^ restricted the adverse outcome to bacteraemia, arguably the MDI of most concern in the routine presentation of fever with neutropenia, and demonstrated their C-statistic was importantly higher (0.71 for bacteraemia vs 0.64 for ‘all’ MDI). The data collected for this meta-analytic validation did not allow us to extract the bacteraemia alone in these sources, and so we cannot replicate this analysis. This means the definition of MDI encompasses life-threatening *Klebsiella* sp septicaemia alongside the incidental detection of rhinovirus shedding.

This study demonstrates that the PICNICC rule at admission does not effectively predict risk of infection or clearly allow discrimination between high and low-risk groups of children and young people presenting with fever and neutropenia when assessed across multiple locations, even after simple recalibrations have been undertaken. The great variation between the initial and subsequent estimates for a ‘malignancy type’ predictor suggests continuing to use this, despite being a clinical heuristic for risk of infection, is probably unhelpful in building a model predicting infection. This may be understood as an effect of the drift of treatments over time, as the ‘malignancy type’ is likely to be a composite of risk of immunosuppression from the disease (in some cases) along with the chemotherapy agents, with their own different propensity to cause mucosal injury. Individual episode-related features connected to the signs of systemic inflammatory response at the presentation of each episode and estimates of immune suppression or barrier disruption, such as the presence of a tunnelled central line, if it is fully implanted or not, or degree of observed mucositis may be of greater consistent value. These features showed some predictive utility during the development of the PICNICC model.^21^

The use of a prediction model has theoretical advantages, allowing a greater degree of discussion with patients and families in sharing a decision to undertake ambulatory management, compared with the bald categorisation as an episode as ‘low’ or ‘high’ risk. Conversely, it may introduce practical barriers to effective implementation, with the requirement for more extensive consultation and the challenge of how patients, families and healthcare workers manage the uncertainty introduced by a predicted risk being discussed. While the PICNICC model appears unhelpful, it remains to be evaluated if alternative prediction models, such as the method proposed by Esbenshade *et al* for non-neutropenic fever,^25^ prove robust in re-evaluation. Caution should be exercised by those wishing to build new prediction models however, as many models are built poorly, for no good purpose, and never validated,^26^ and following guidance such as the TRIPOD statement will help.^27^

The unique PICNICC Collaboration has enabled such extensive evaluations to take place and redirect efforts into evaluation of other systems of immediate stratification, or alternative approaches for the rational management of fever with neutropenia in children, such as biomarker-guided reduction in antibiotic duration or ‘day two’ risk stratification.

Addressing the issues of validating a risk prediction model, when coupled with further studies investigating the utility of biomarkers and exploring how predictive information could be used by children, young people and their families in making decisions about the treatment of fever with neutropenia will allow us to personalise our treatment more effectively, and develop pragmatic trials to improve management of fever with neutropenia. This meta-analysis shows it would be inappropriate to use the PICNICC model at admission as the basis of a clinical trial, but further work should focus on integrating these and other available data sets to develop and then validate alternative prediction models and decision rules.

## References

[1] Wicki S, Keisker A, Aebi C, et al Risk prediction of fever in neutropenia in children with cancer: a step towards individually tailored supportive therapy? Pediatr Blood Cancer 2008;51:778–83. 10.1002/pbc.21726 18726920

[2] Duncan C, Chisholm JC, Freeman S, et al A prospective study of admissions for febrile neutropenia in secondary paediatric units in South East England. Pediatr Blood Cancer 2007;49:678–81. 10.1002/pbc.21041 17066460

[3] El-Mahallawy HA, El-Wakil M, Moneer MM, et al Antibiotic resistance is associated with longer bacteremic episodes and worse outcome in febrile neutropenic children with cancer. Pediatr Blood Cancer 2011;57 10.1002/pbc.22926 21671364

[4] Kamboj M, Sepkowitz KA Nosocomial infections in patients with cancer. Lancet Oncol 2009;10:589–97. 10.1016/S1470-2045(09)70069-5 19482247

[5] Speyer E, Herbinet A, Vuillemin A, et al Agreement between children with cancer and their parents in reporting the child's health-related quality of life during a stay at the hospital and at home. Child Care Health Dev 2009;35:489–95. 10.1111/j.1365-2214.2009.00972.x 19638023

[6] Sung L, Feldman BM, Schwamborn G, et al Inpatient versus outpatient management of low-risk pediatric febrile neutropenia: measuring parents' and healthcare professionals' preferences. JCO 2004;22:3922–9. 10.1200/JCO.2004.01.077 15459214

[7] Phillips RS, Sutton AJ, Riley RD, et al Predicting infectious complications in neutropenic children and young people with cancer (IPD protocol). Syst Rev 2012;1:8 10.1186/2046-4053-1-8 22588015 PMC3351734

[8] Phillips RS, Lehrnbecher T, Alexander S, et al Updated systematic review and meta-analysis of the performance of risk prediction rules in children and young people with febrile neutropenia. PLoS One 2012;7:e38300 10.1371/journal.pone.0038300 22693615 PMC3365042

[9] Leontiadis G, Sreedharan A, Dorward S, et al Systematic reviews of the clinical effectiveness and cost-effectiveness of proton pump inhibitors in acute upper gastrointestinal bleeding: PPi therapy in patients with endoscopically documented acute bleeding from a peptic ulcer. Health Technology Assessment 2007;11:15–39. 10.3310/hta11510 18021578

[10] Miedema KGE, de Bont ESJM, Oude Nijhuis CSM, et al Validation of a new risk assessment model for predicting adverse events in children with fever and chemotherapy-induced neutropenia. JCO 2011;29:e182–4. author reply e85 10.1200/JCO.2010.32.7767 21245423

[11] Phillips B, Ranasinghe N, Stewart LA, et al Ethical and regulatory considerations in the use of individual participant data for studies of disease prediction. Arch Dis Child 2013;98:567–8. 10.1136/archdischild-2013-304149 23661573

[12] Haeusler GM, Phillips RS, Lehrnbecher T, et al Core outcomes and definitions for pediatric fever and neutropenia research: a consensus statement from an international panel. Pediatr Blood Cancer 2015;62:483–9. 10.1002/pbc.25335 25446628

[13] Goldstein B, Giroir B, Randolph A, et al International pediatric sepsis consensus conference: definitions for sepsis and organ dysfunction in pediatrics*. Pediatr Crit Care Med 2005;6:2–8. 10.1097/01.PCC.0000149131.72248.E6 15636651

[14] Collins GS, Ogundimu EO, Altman DG Sample size considerations for the external validation of a multivariable prognostic model: a resampling study. Stat Med 2016;35:214–26. 10.1002/sim.6787 26553135 PMC4738418

[15] Altman DG, Royston P What do we mean by validating a prognostic model? Stat Med 2000;19:453–73. 10.1002/(SICI)1097-0258(20000229)19:4&lt;453::AID-SIM350&gt;3.0.CO;2-5 10694730

[16] Steyerberg EW Clinical prediction models: a practical approach to development, validation, and updating. New York: Springer, 2009.

[17] Debray TPA, Damen JAAG, Snell KIE, et al A guide to systematic review and meta-analysis of prediction model performance. BMJ 2017;356 10.1136/bmj.i6460 28057641

[18] Phillips RS, Sung L, Ammann RA, et al Erratum: predicting microbiologically defined infection in febrile neutropenic episodes in children: global individual participant data multivariable meta-analysis. Br J Cancer 2016;114:e17 10.1038/bjc.2016.137 27228292 PMC4984466

[19] Toll DB, Janssen KJM, Vergouwe Y, et al Validation, updating and impact of clinical prediction rules: a review. J Clin Epidemiol 2008;61:1085–94. 10.1016/j.jclinepi.2008.04.008 19208371

[20] Snell KIE, Hua H, Debray TPA, et al Multivariate meta-analysis of individual participant data helped externally validate the performance and implementation of a prediction model. J Clin Epidemiol 2016;69:40–50. 10.1016/j.jclinepi.2015.05.009 26142114 PMC4688112

[21] Atlas C, Aad G, Abbott B, et al Identification of boosted, hadronically decaying W bosons and comparisons with ATLAS data taken at [Formula: see text] TeV. Eur Phys J C Part Fields 2016;76. 10.1140/epjc/s10052-016-3978-z PMC4946871 27471432

[22] Klastersky J, Paesmans M, Rubenstein EB, et al The multinational association for supportive care in cancer risk index: a multinational scoring system for identifying low-risk febrile neutropenic cancer patients. J Clin Oncol 2000;18:3038–51. 10.1200/JCO.2000.18.16.3038 10944139

[23] Ammann RA, Bodmer N, Hirt A, et al Predicting adverse events in children with fever and chemotherapy-induced neutropenia: the prospective multicenter SPOG 2003 FN study. JCO 2010;28:2008–14. 10.1200/JCO.2009.25.8988 20231680

[24] Haeusler GM, Thursky KA, Mechinaud F, et al Predicting infectious complications in children with cancer: an external validation study. Br J Cancer 2017;117:171–8. 10.1038/bjc.2017.154 28609435 PMC5520507

[25] Esbenshade AJ, Pentima MCD, Zhao Z, et al Development and validation of a prediction model for diagnosing blood stream infections in febrile, non-neutropenic children with cancer. Pediatr Blood Cancer 2015;62:262–8. 10.1002/pbc.25275 25327666 PMC4402108

[26] Wynants L, Collins GS, Van Calster B Key steps and common pitfalls in developing and validating risk models. BJOG: Int J Obstet Gy 2017;124:423–32. 10.1111/1471-0528.14170 27362778

[27] Moons KGM, Altman DG, Reitsma JB, et al Transparent reporting of a multivariable prediction model for individual prognosis or diagnosis (TRIPOD): explanation and elaboration. Ann Intern Med 2015;162:W1–73. 10.7326/M14-0698 25560730

