# Supplementary material for: Individual participant data validation of the PICNICC prediction model for febrile neutropenia
Source: Arch Dis Child. 2019 Nov 5;105(5):439–45. doi: 10.1136/archdischild-2019-317308 (PMC7212933; doi:10.1136/archdischild-2019-317308)
Supplement: Supplementary data [file archdischild-2019-317308supp001.pdf]

APPENDIX ONE

Table: Distribution of linear predictors

Study	No MDI					MDI Identified				
	Mean	SD	Median	Minimum	Maximum	Mean	SD	Median	Minimum	Maximum
liverpool	3.457	0.857	3.335	1.745	5.844	4.001	1.191	3.634	2.326	5.834
Sheffield	3.085	8.694	1.983	-13.689	37.440	3.622	8.724	1.947	-13.378	37.771
nottingham	2.275	2.891	2.693	-11.420	5.057	0.713	5.425	2.777	-11.952	4.244
leeds	4.517	2.463	3.682	1.919	11.955	3.904	1.014	4.357	2.174	4.962
melb101	3.903	1.819	3.501	0.956	12.060	5.193	2.536	4.821	2.385	12.088
Bauters	2.178	0.855	1.986	0.527	3.450	2.454	0.795	2.741	1.196	3.187
Melb650	3.301	2.296	3.585	-11.499	6.674	3.878	1.971	4.036	-12.575	7.295

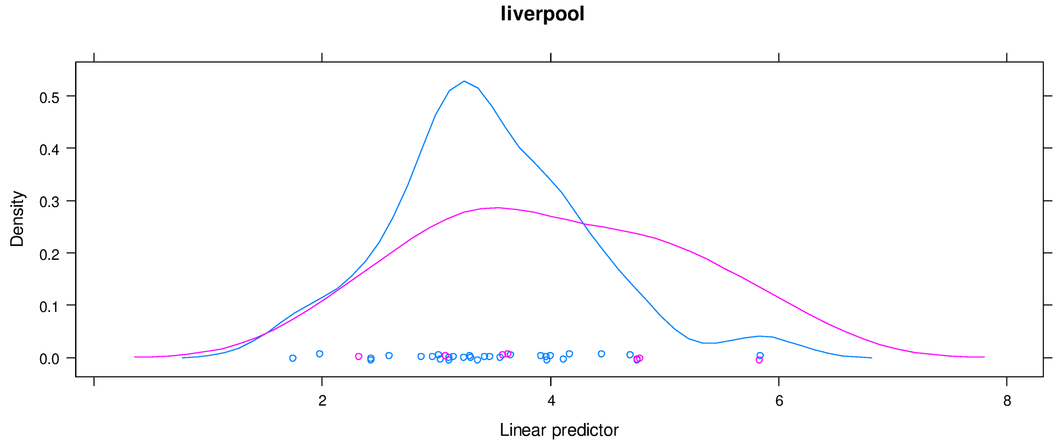

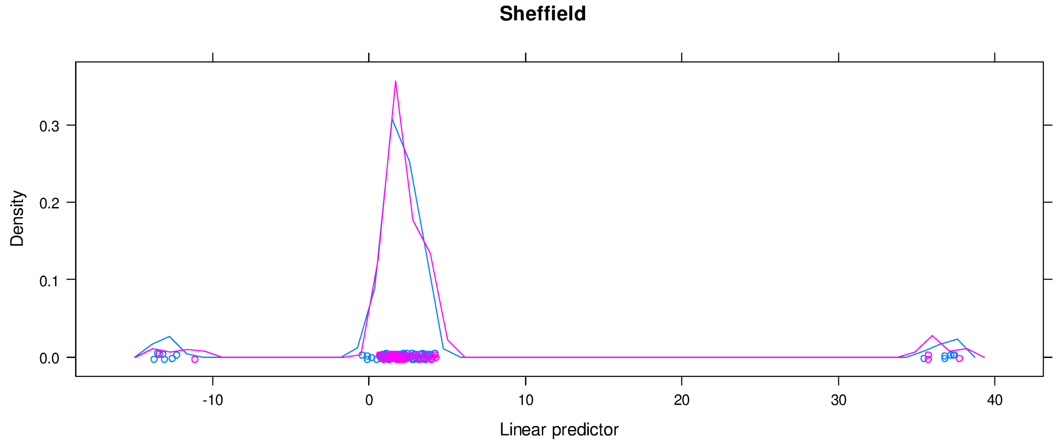

Phillips B, *et al. Arch Dis Child* 2020; 105:439–445. doi: 10.1136/archdischild-2019-317308

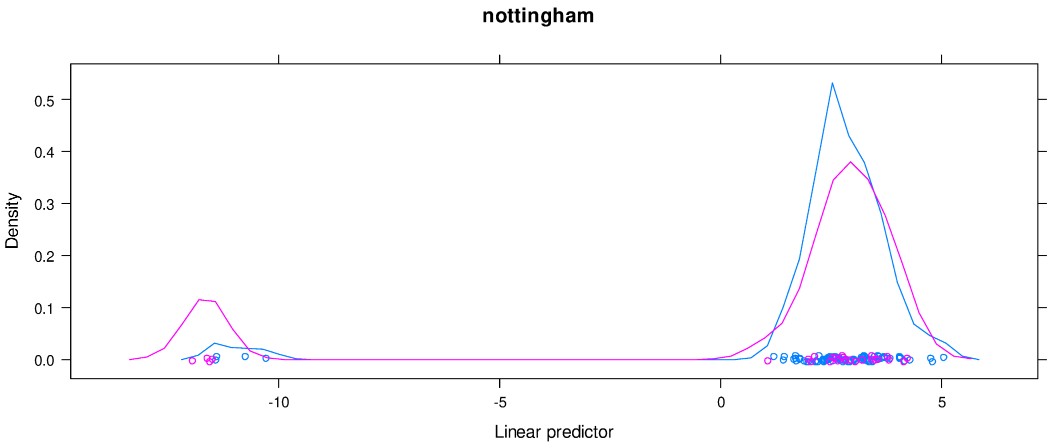

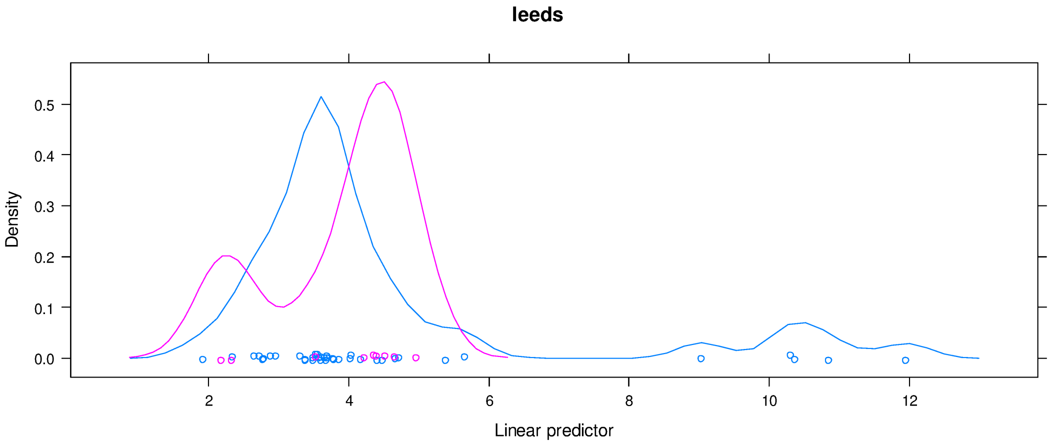

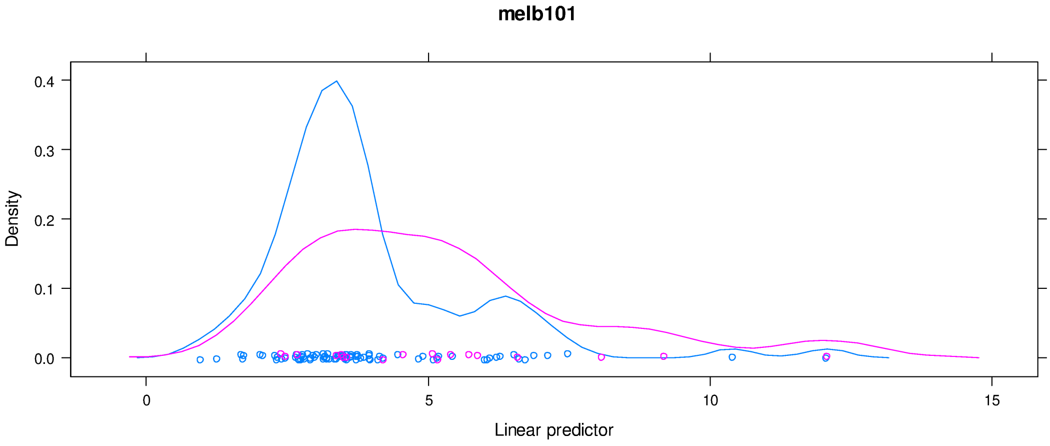

Phillips B, *et al. Arch Dis Child* 2020; 105:439–445. doi: 10.1136/archdischild-2019-317308

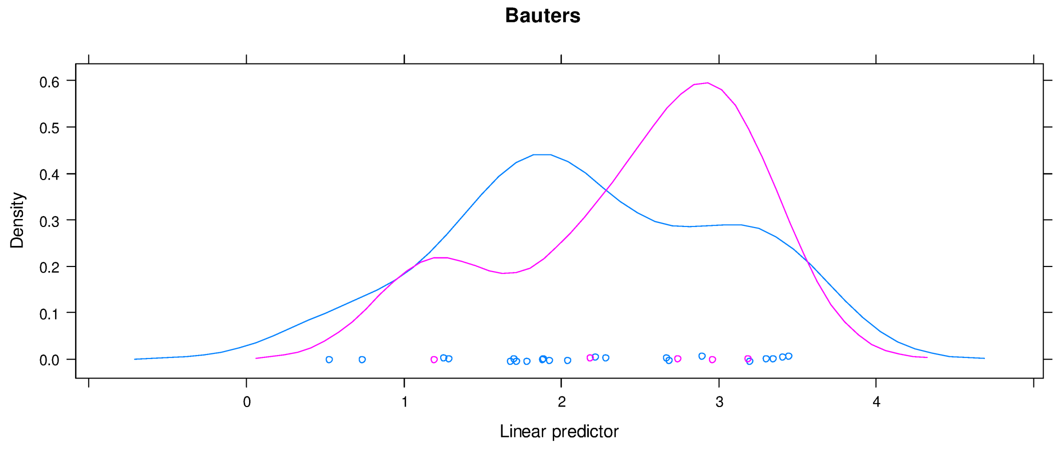
Bauters
Density
0.6
0.5
0.4
0.3
0.2
0.1
0.0
0
1
2
3
4
Linear predictor

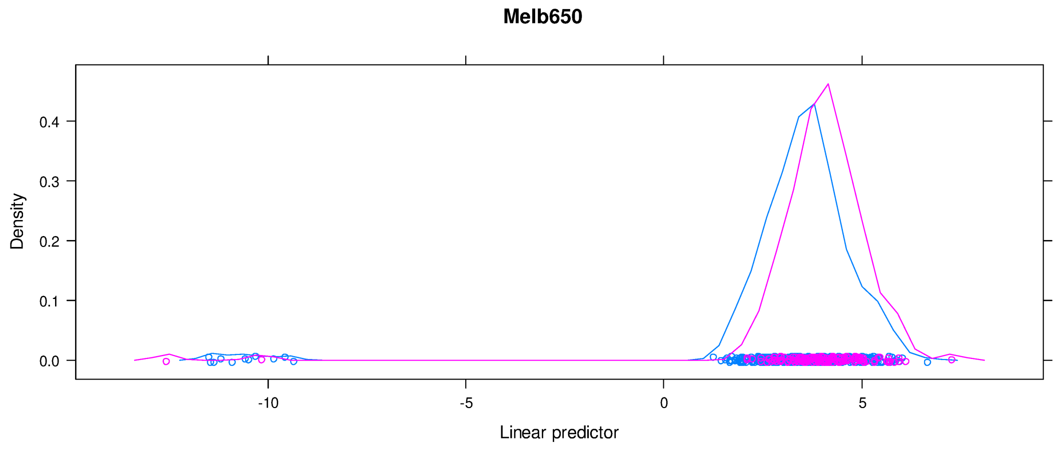
Melb650
Density
0.4
0.3
0.2
0.1
0.0
-10
-5
0
5
Linear predictor

APPENDIX TWO

Calibration plots of unadjusted values

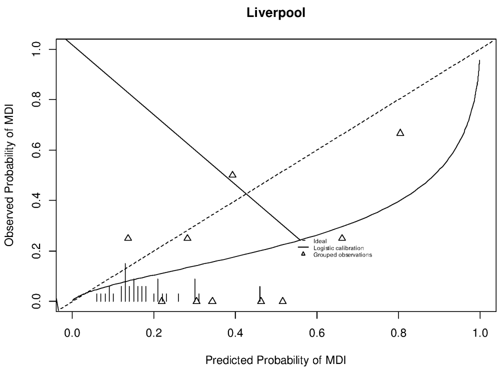

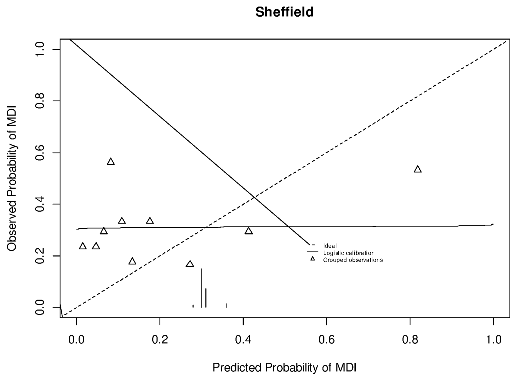

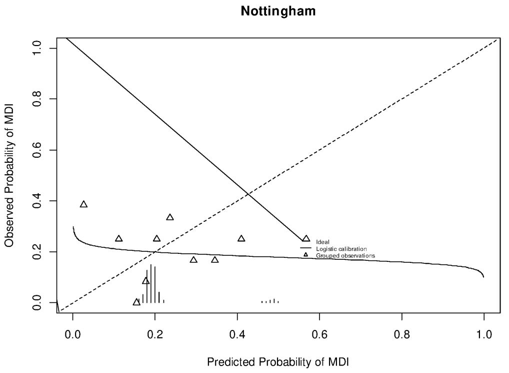

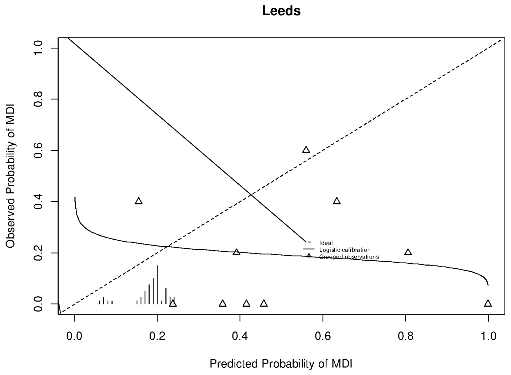

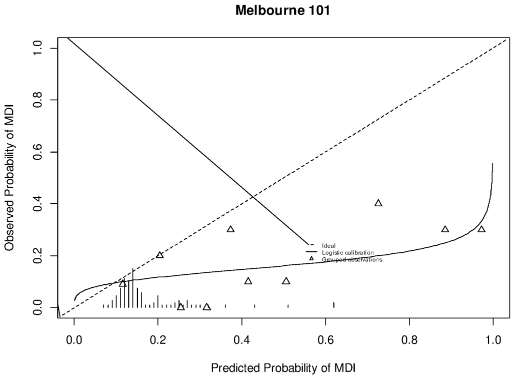

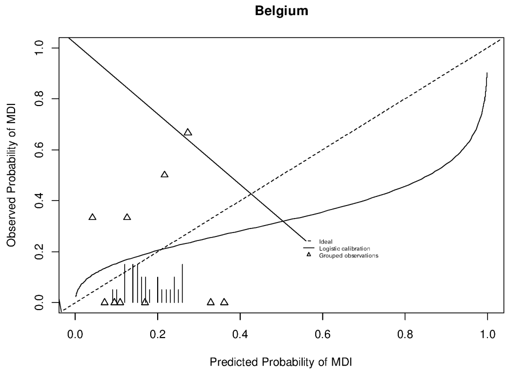

Phillips B, *et al. Arch Dis Child* 2020; 105:439–445. doi: 10.1136/archdischild-2019-317308

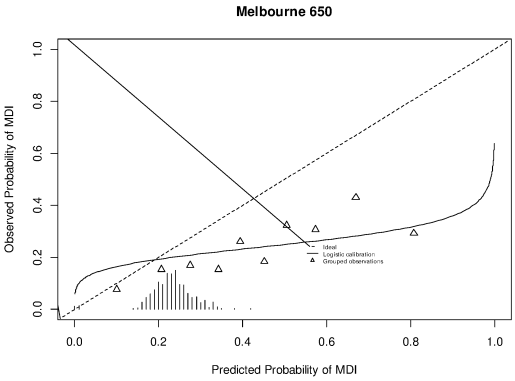
Melbourne 650
Observed Probability of MDI
Predicted Probability of MDI
0.0
0.2
0.4
0.6
0.8
1.0
Ideal
Logistic calibration
Grouped observations

UNIVERSITY *of York*

APPENDIX THREE

Selected estimates of performance: Proportionate MDI intercept change

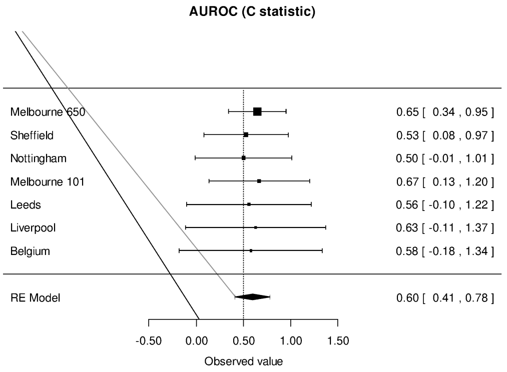

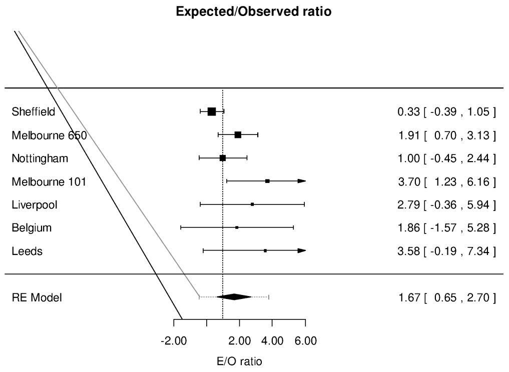

`EO 1.67 (95% CI 0.65-2.70, 95% PrI -0.43to3.78) tau sq 0.87`

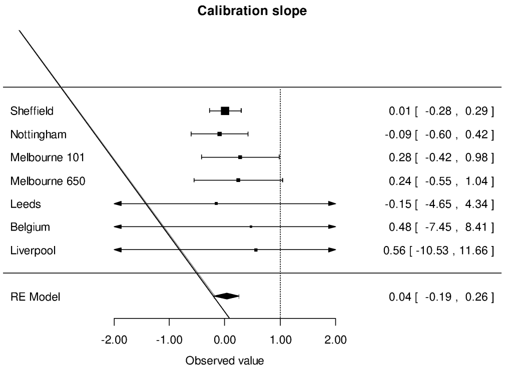

Selected estimates of performance: Interpolated MDI intercept change

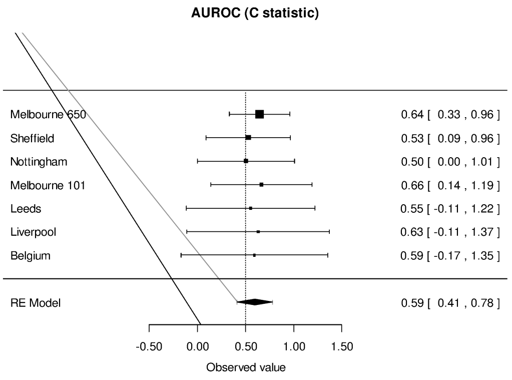

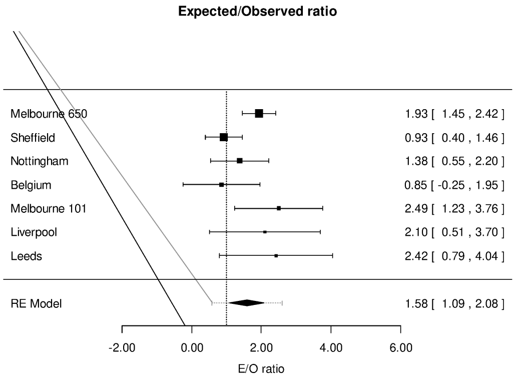

`E/O 1.58 (95% CI 1.09to2.08, 95% CrI 0.57to2.59) tausq 0.20`

Phillips B, *et al. Arch Dis Child* 2020; 105:439–445. doi: 10.1136/archdischild-2019-317308

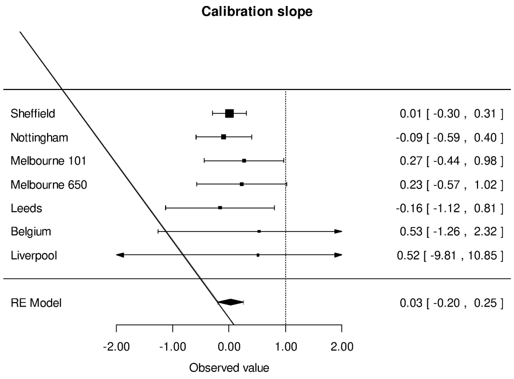

Phillips B, *et al. Arch Dis Child* 2020; 105:439–445. doi: 10.1136/archdischild-2019-317308
